# The biopsychosocial model and chiropractic: a commentary with recommendations for the chiropractic profession

**DOI:** 10.1186/s12998-017-0147-x

**Published:** 2017-06-07

**Authors:** Jordan A. Gliedt, Michael J. Schneider, Marion W. Evans, Jeff King, James E. Eubanks

**Affiliations:** 1 College of Chiropractic, Logan University, Chesterfield, MO USA; 2 Department of Physical Therapy, University of Pittsburgh, Pittsburgh, PA USA; 3 Department of Food Science, Nutrition, and Health Promotion, Mississippi State University, Starkville, MS USA; 4 Medical College of Wisconsin, Department of Neurosurgery, Milwaukee, WI USA; 5 Brody School of Medicine, East Carolina University, Greenville, NC USA

**Keywords:** Biopsychosocial, Biomedical, Chiropractic, Back pain, Neck pain, Pain management

## Abstract

There is an increasing awareness, interest and acceptance of the biopsychosocial (BPS) model by all health care professionals involved with patient care. The areas of spine care and pain medicine are no exception, and in fact, these areas of health care are a major centerpiece of the movement from the traditional biomedical model to a BPS model of patient assessment and delivery of care. The chiropractic approach to health care has a history that is grounded in key aspects of the BPS model. The profession has inherently implemented certain features of the BPS model throughout its history, perhaps without a full understanding or realization. The purpose of this paper is to present an overview of the BPS model, its relationship with spine care and pain management, and to discuss the BPS model, particularly psychosocial aspects, in the context of its historical relationship with chiropractic. We will also provide recommendations for the chiropractic profession as it relates to successful adoption of a full integration of the BPS model.

## Background

Biological influences are an important component of health and disease, particularly in the area of spine care and pain management. Acknowledging this aspect of patient presentation holds value in guiding appropriate management approaches. Appreciation of the psychological and social aspects of patient care carries significance as well and should not be overlooked. The objective of this paper is to provide an overview of the biopsychosocial (BPS) model and predominantly the psychosocial components of spine care, pain management, and the chiropractic profession. The authors recognize the importance of the biological element of health and disease, particularly in the fields of spine and pain care. The central focus of this paper, however, is on the psychological and to a lesser extent social aspects because, as illustrated in this paper, these are not fully appreciated in chiropractic at this time.

### The biopsychosocial model

The notion of psychological and social determinants contributing to the development, persistence and healing of illness is not novel. Discussions of psychosocial influences in health and disease have been noted for multiple centuries [1, 2]. A noteworthy example is Francis Peabody’s famous speech, “The Care of the Patient”, given to attendees of Harvard Medical School in 1927 which specifically addressed the importance of the art of patient-centered medicine that extends beyond the impersonal scientific mechanisms of the treatment of disease [3]. Interestingly, this dialogue is reflective of chiropractic philosophy which has regularly emphasized the art and science of chiropractic care which includes a whole person approach that aims to investigate, eliminate, and prevent the cause of disease.

Despite historical considerations of the potential for psychosocial impact on physical well-being, the majority of the twentieth century was governed by the biomedical model. This model, also known as the pathoanatomic model, resulted from Virchow’s deduction that all disease results from cellular aberration [4]. This mechanistic paradigm played a crucial role in the successful eradication of numerous infectious diseases, with the result of significant extension in life expectancy seen in the twentieth century. Since many biomedically-responsive problems have now been addressed successfully, health care as a whole has begun to turn its attention to quality of life issues that require a new model of care.

**Fig. 1:**
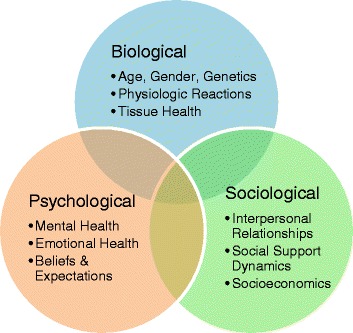
[2] An illustration of the biopsychosocial model comprised of biological, psychological, and sociological influences

The last few decades have seen considerable attention and acceptance of psychosocial influences in health, exemplified by the BPS model [5]. The development of the BPS model is attributed to Engel’s challenge of the biomedical model in 1977 [6]. Engel argued that the biomedical model leaves no room for appreciation of psychosocial considerations in disease and therefore “distorts perspectives and even interferes with patient care” [6]. Engel proposed a new medical model that intertwined biological, psychological, and sociological factors: physical or chemical alterations to the body (biological factors), personal development and psychological/mental health factors, and social determinants [6] (Fig. 1).

**Fig. 2:**
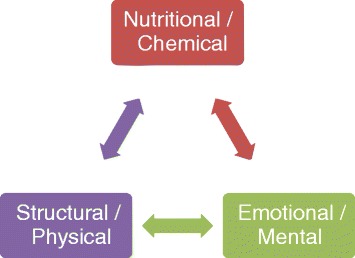
[8] The “triad of health” illustrating the approach to chiropractic health care

According to this model, any element of human function can have an effect on any other element [2, 7]. Therefore, all aspects of human illness can be characterized by a confluence of biological, psychological, behavioral, and social connections [2]. The chiropractic approach has many traditional characteristics that are also common to the BPS model of care. Illustrations showing the chiropractic approach that highlights the interdependent nature of mental/emotional, biochemical, and structural influences on health are commonplace and have been discussed in chiropractic teachings for several years (Fig. 2) [8]. Chiropractors have also traditionally connected with patients’ socioemotional status and met their patients with an egalitarian relationship that includes patience, attention, kindness, and sympathy [9, 10]. This exemplifies, in a rudimentary sense, an awareness of psychosocial determinants of health and recovery as a highlighted feature of chiropractic teachings.

### Spine pain, chiropractic, and the biopsychosocial model

Pain and associated disability are growing health concerns that demand continued evaluation to identify optimal prevention and management approaches. Point prevalence of chronic pain among adults has been measured at up to 41% of the population in both developed and developing countries [11]. In the United States chronic pain is estimated to affect more than 100 million adults, producing over $600 billion dollars of direct and indirect costs each year [12]. Low back and neck pain, specifically, account for the greatest cause of disability in both men and women in most countries worldwide [13]. As chronic pain and disability has increased to epidemic proportions, conventional patient care provided by a solo practitioner has become de-valued, leading to an emphasis toward a team-based system. Included in this shift in ideology is a growing interest in the BPS model, which is now the dominant model to explain and manage pain [14].

Beyond biological influences, there are many psychosocial factors that have been shown to negatively contribute to heightened pain awareness and disability. These factors include: fear-avoidance beliefs, depression and anxiety, post-traumatic stress disorders, unsupportive social and interpersonal relationships, catastrophizing thoughts, low levels of self-efficacy, and maladaptive beliefs [14]. This medley of psychosocial factors can contribute through multiple inputs toward a continuous cycle of persistent pain.

**Fig. 3:**
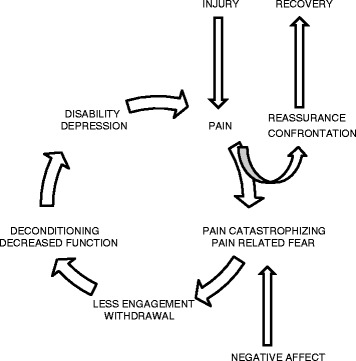
[14] The Fear-Avoidance Model is an example of the interconnectedness between biological and psychosocial influences that may contribute to persistent pain

For example, a pain experience may lead to catastrophizing thoughts and a subsequent fear of specific movements or a more general avoidance of all physical activity. Fear of movement and avoidance of physical activity may lead to a social and physical withdrawal. This withdrawal may produce mental and emotional depression or anxiety, which can in turn directly intensify the individual’s pain experience and add to the potential for disability (Fig. 3). This cycle of pain and dysfunction can be further perpetuated by a clinician’s focus on pathoanatomical diagnoses and reliance on passive treatments. The authors postulate that without often recognizing it, chiropractors have historically been early innovators at discussing and confronting the basics of the fear-avoidance model and implementing strategies to break and prevent recurrence of this cycle. It is the authors’ experiences that for decades many chiropractors have implemented practices such as reassurance, advice to avoid bed rest, efforts toward early activation, graded exposure and return to normal movements despite pain.

Field et al. investigated components of the fear-avoidance model in patients with low back pain and examined change in these measures pre and post-initial chiropractic visit. Measurements included patient self-reported pain intensity, perceived self-efficacy, fear-avoidance behavior, catastrophizing, and beliefs surrounding back pain. Most patients that presented to an initial chiropractic visit with higher scores of psychological distress showed a decrease in these scores after a few days post visit. The investigators suggested that something other than physical treatment, such as active listening, providing plausible explanations for pain, and providing reassurance may have accounted for some of the improvement seen [15].

Engaging in positive psychosocial dynamics and interventions may play a role in reducing the risk for developing chronic pain and disability, but also in developing resilience toward chronic pain [14, 16, 17]. For those who experience chronic pain, studies have shown that consideration of these psychosocial factors aids in the understanding that individuals can live with chronic pain without concurrently developing disability [14, 18]. It appears that active coping techniques are an important strategy in developing chronic pain resilience [14]. These active coping strategies include many components of chronic pain related cognitive behavioral therapy (CBT) [19]. These may include engaging in positive thinking, re-directing negative self-thoughts and statements toward positive self-thoughts and affirmations, engaging in pain distracting activities, carrying out as much physical exercise as able within pacing parameters, and utilizing active relaxation exercises and stretching [14]. Additional positive psychosocial interventions include: Acceptance and Commitment Therapy (ACT), mindfulness meditation, and cognitive-behaviorally-oriented educational interventions such as “Explaining Pain” sessions. All of these interventions are backed by encouraging evidence to support inclusion of these types of self-empowering strategies in the management of pain related complaints [14].

Inclusion of psychosocial oriented methods within a multimodal treatment approach has been shown to produce superior outcomes in chronic pain patients compared to unimodal treatments [20, 21]. These superior results have led to these multimodal approaches being embraced in clinical practice guidelines. Monticone and colleagues conducted a recent randomized controlled trial to evaluate the effect of a group-based rehabilitation program that combined multimodal exercises with CBT versus general physiotherapy exercise for patients with chronic neck pain. Significantly greater improvements were seen, which remained at 12 month follow up, in the multimodal rehabilitation group compared to the physiotherapy only group. Additionally, the physiotherapy group did not show any post-intervention reduction in measured kinesiophobia or catastrophizing [20]. A 2017 review of evidence of noninvasive treatments for acute, subacute, and chronic low back pain as part of an American College of Physicians clinical practice guideline recommended that clinicians and patients should initially select nonpharmacologic therapies. The guideline recommendations support inclusion of mindfulness-based stress reduction, progressive relaxation, CBT, exercise, yoga, tai chi, spinal manipulation, and multidisciplinary rehabilitation as first choice options [21].

The shared aims of all psychosocial interventions are to galvanize active patient involvement in healthy lifestyle behavior, empower the patient to lead a lifestyle that holds greater self-efficacy, and to reduce the need for dependence on health care providers’ treatments. Chiropractic teachings have traditionally stressed the concept of the body as a self-healing mechanism and de-emphasized the need for foreign interventions, such as drugs and surgery [22, 23]. B.J. Palmer, who is often considered the “developer” of chiropractic, coined the term “above-down-inside-out” to refer to the notion that the innate intelligence of the body controls healing from inside out [23] and chiropractic intervention merely assists the body to optimize and achieve self-healing. Palmer explicitly stated that “over-adjusting is kept to a bare minimum if at all” [10]. These are examples of messages conveyed by chiropractors that encouraged a reliance on the power of self-healing [22, 23] and inherently implies self-efficacy. Although originally attributed to A.T. Still, Clarence Gonstead, a notable chiropractor whose teaching remains influential in the present day, regularly discussed the notion that upon finding the need to manipulate the spine one should fix it and leave it alone [24, 25]. This discussion held an implication that the body has an intrinsic capability to heal itself, and when the underlying obstruction is fixed, the body will complete the process and no further intervention is necessary [24].

Chiropractors also have long been advocates for active healthy lifestyle modifications such as nutritional/dietary counseling, exercise enhancement, and stress reduction strategies [8, 9, 22, 26, 27]. Palmer reported that a chiropractic clinic is premised on two “vital principles”. One of these principles included rehabilitation which cannot be done externally through means such as manipulation, but instead by internal use of the patient [27]. Instead of waiting for symptoms to appear or become advanced, chiropractors have also maintained a focus on early intervention and prevention measures that include addressing both biological and psychosocial elements [9, 26, 28–32].

Recent studies have shown chiropractors’ abilities to effectively influence patients toward healthy lifestyle behaviors [33–35]. Coulter previously reported on practitioner-patient relationships and paradigms in health care and noted a review by Coulehan which described typical chiropractic encounters including distinct elements that involve “a plan that requires patient commitment and cooperation” and a goal to “develop a positive image of personal control over one’s health [33].”

Evans has proposed a method of providing positive advice by use of the “ABCS’”, a mnemonic of wellness and health promotion methods in chiropractic practice [36]. In this case the “A” is to assess the overall health and wellness needs of every patient beyond pain management. The “B” is to extol the benefits of positive behavior change to the patient. The “C” is to use routine chiropractic visits to launch positive lifestyle changes on patients rather than focusing on a single visit that acutely addresses an episode of heightened pain. The “S” is to stay the course when the patient has begun to make successful changes or is frustrated with lack of progress.

The National Board of Chiropractic Examiners periodically conducts a national survey of the profession, which has indicated that advice on increasing physical activity and healthy diet is a common strategy utilized in general chiropractic practice [37]. A study of chiropractic interns and clinicians in a teaching clinic found that interns can successfully assess and advise tobacco users on cessation and provide necessary information to route them into cessation programs [34].

Ndetan and colleagues assessed the National Health Interview Survey data in 2009 and isolated those receiving spinal manipulation from respondents in the survey who had traditional medical care. When asked if they had tried to make changes in behavior at the advice of their chiropractor or osteopath, they reported an attempt to make changes in their health care behavior greater than 85% of the time. This was virtually identical to the percent who reported attempting behavioral change based on the advice of a conventional medical physician, who might be more likely to render advice on general health measures [35].

### Words matter

Without always realizing it, the chiropractic profession has been a pioneer in spine care and pain management by employing aspects of a whole-person BPS approach to health care [8–10, 22, 23, 27, 28] with an emphasis on self-healing [38]. This approach has involved methods that encompass psychosocial, emotional/spiritual, physical, and healthy lifestyle components [8–10, 22, 26–28, 38–40] which may promote functional gain/preservation, reduction of pain interference and maximization of quality of life. Nevertheless, there are two sides to every coin. Although well-intentioned, some characteristics of typical chiropractic-patient engagement practices may be inadvertently negating positive influences or even unwittingly feeding into patients’ maladaptive thoughts or beliefs. The words that any health care provider says and the way in which they are delivered to patients are very important in influencing positive or negative outcomes. Although the chiropractor’s motivations may be well-intended, practices such as providing reports of findings that over-emphasize pathoanatomical diagnoses or utilizing disability and pain associated language can induce negative, disempowering beliefs and behavior [14, 41–43]. Other chiropractic practices may lower patients’ self-efficacy and promote heightened fear, including: presentation of diagnostic theories such as the “bone out of place” theory that requires routine correction, and recommending indefinite maintenance care and long treatment plans built around passive care. Patients’ self-efficacy can also be lowered by utilization of communication styles that inherently involves fear tactics or “loss-framing” whereby messages emphasize costs or losses to health if action is not taken to justify the need for ongoing passive treatments.

The unintended consequence of engaging in these communication strategies with patients is that they convey an underlying message to the patient that if they miss a chiropractic treatment session their condition will worsen or persist. This concept promotes reliance on the chiropractor, which innately strips them of their abilities to enhance their well-being and creates a co-dependent doctor-patient relationship. How often in a chiropractic office does one hear a patient with benign non-specific spine complaints present for care because their “back went out of alignment”? Instead of educating this patient about the benign nature of non-specific spine pain and re-directing their attention toward proper self-care management strategies, this patient has received a message that has created a dependence on the health care practitioner. Essentially, the patient is led to believe that s/he has no control (loss of self-efficacy) over his/her condition.

Reports of findings to patients that concentrate on benign imaging findings and stress the need for continual manipulative therapy can slow down recovery. These concepts serve to reinforce the idea that positive physical, mental, emotional, and social self-care strategies are inadequate, and the patient must rely on an indefinite future of chiropractic treatments to maintain health. This approach distracts from the concepts of active coping or self-efficacy. Instead, it places the patient in a co-dependent chiropractic-patient relationship, characterized by passive coping strategies and fear-avoidance beliefs, both of which are known predictors of persistent disability [14, 44].

It is interesting to note that although positive psychosocial factors and interventions have been shown to reduce individuals’ risk for developing chronic pain and ameliorate chronic pain sufferers’ resilience to disability, the existence and extent of negative psychosocial behaviors may have a greater impact on individuals’ prognosis for persistent pain and disability [45–47]. With this in mind, it is appropriate to ask if the use of these chiropractic communication practices is ultimately creating or feeding into pre-existing negative psychosocial behaviors and unwittingly increasing the risk of pain and disability. As health care practitioners, we need to be consciously aware of the potentially negative consequences of the words we say in a doctor-patient encounter, particularly during a report of findings. A helpful self-inquiry may be, “Do my communication practices with patients relay a positive self-empowering message, or one of unnecessary dependence?”

### Positive motivations in patient communications

Message framing is of key importance in any doctor-patient encounter. Some practice management strategies use fear of a “deadly subluxation” as “silent killers” or illustrations of the “phases of spinal degeneration” to motivate patients to attend long-term chiropractic treatment plans. Instead of these fear tactics, “gain-framing” (messages that emphasize benefits or gains) a message has been shown to better motivate patients to make positive changes in health behaviors [48]. This is basically explaining the positive aspects of care and behavior changes, versus emphasizing the negative aspects of not making changes or following through with recommendations. While experts acknowledge that fear can be a powerful motivator, in many cases it is unethical to scare patients with the intent to coerce care or improve compliance with chiropractic treatment plans. The use of scare tactics has been shown to negatively influence individuals’ behaviors, especially those with low levels of perceived self-efficacy [49, 50]; this reinforcement of negative behavior in those with low self-efficacy can feed into pre-existing fears of the patient, create further negative expectations of the future, and foster catastrophizing thoughts [14]. This negative communication style is less effective than motivating patients to change using positive language that emphasizes benefits of health behavior modification.

The Health Belief Model suggests that not only must self-efficacy be enhanced for successful behavior change, but the perceived benefits of changing a behavior must outweigh any negatives or barriers [51]. This is the rationale for careful reports of findings and the reiteration of those findings as care continues. Stressing the positive changes that are likely to occur rather than holding patients hostage to a rigid treatment plan, is likely to facilitate the type of doctor-patient partnership that is truly needed to help them get well and stay well.

Motivational interviewing (MI), CBT, and ACT can be used as preferred techniques for chiropractors to initiate doctor-patient communication regarding instituting self-empowerment strategies. These techniques can provide opportunities to structurally integrate positive communication regarding behavioral change into a typical chiropractic session and are malleable to single visit use or, optimally, in a series of visits which may coincide with a short course of multi-visit sessions often implemented in a trial of care. These techniques are not exclusive to other options that may be incorporated into chiropractic visits and more research is needed to further asses the utility of these kinds of techniques within the context of chiropractic care.

MI can assist in identifying targeted behavioral change, motivation and obstacles in a patient-centered discussion. MI may be particularly applicable to the practicing chiropractic clinician because of its specific design for primary care settings where time is a limiting factor. The underlying concept of MI allows for patient autonomy and is based on four principles: 1) expressing empathy for the patient and their health issue, 2) developing discrepancy between what needs to occur for positive changes and perhaps what the patient is expressing or willing to do, 3) “rolling with resistance” when the patient expresses negativity in their ability to make needed changes, and 4) supporting self-efficacy such that the patient understands that when they are ready to make a change, the doctor is ready and willing to support them with the needed behavior changes [52].

CBT is another commonly used, time-limited psychotherapeutic intervention that has shown to be valuable across a multitude of mental and behavioral conditions, including chronic pain related ailments. CBT is described as a structured approach that concentrates on the relationships between thoughts, emotions, and behaviors. CBT is rooted in the development of a robust therapeutic relationship that nurtures patient development and use of active, problem-solving skills which can be applied to actively manage the challenges associated with chronic pain [19].

ACT can also be employed as a method to assist in shifting one’s perspective or positively deal with personal experiences. ACT is based on functional contextualism and Relational Frame Theory. ACT aims to focus on the processes of language that are thought to be involved in psychopathology and its amelioration. ACT includes the viewpoint that attempting to change problematic thoughts and feelings as a means of coping can be disparaging; however, constructive alternatives such as acceptance, mindfulness, cognitive defusion, and committed action may be of value [53].

### Recommendations

It is evident that how health care providers interact with patients and the communication style they employ are of immense importance. The messages conveyed through a doctor-patient interaction, both directly and indirectly, can affect patients in either a positive empowering way that stimulates personal growth and self-efficacy or in a negative way that creates/re-enforces self-limiting behavior and passivity [14, 45–47]. It is with great honor that chiropractors can play such a monumental role in the health and well-being of patients who seek chiropractic care. With this honor comes great responsibility as well. It is this responsibility that requires chiropractors to continuously evaluate habits, practices and communication efforts and refine these mannerisms to render the highest quality of care and position themselves as expert leaders with the highest level of awareness and competence.

Therefore, it is important that the chiropractic profession hold strongly and continue to promote the foundational tenets of chiropractic that align with the BPS model of care. Efforts to educate students and practicing chiropractors are needed to transition to obtaining a full understanding of methods to evaluate psychosocial influences and provide a full arsenal of methods to implement with an aim to empower patients toward a goal of self-efficacy. Attention should be given toward minimizing the negative communication practices that foster reliance on passive care and maximizing those positive messages of the chiropractic doctor-patient interaction that foster self-efficacy and self-reliance in our patients. A concentration in chiropractic research should be given toward studies investigating the most optimal strategies in implementing the full BPS model of care both within the chiropractic encounter, but also in inter-professional team based environments.

At this time, it appears that educational efforts to address the psychosocial aspects of chiropractic care are limited. The Council on Chiropractic Education (CCE) requires courses in health promotion that includes the “recognition of the impact of biological, chemical, behavioral, structural, psychosocial and environmental factors on general health” as a specified meta-competency for chiropractic programs to address [54]. Unfortunately, the extent of implementation does not appear to be substantial. Possibly the most notable educational effort at the chiropractic college level has occurred at a single chiropractic college within a post-graduate primary spine care practitioner residency program [55]. Murphy has introduced an approach to the primary management of spine related disorders, in his textbook series, in which BPS principles are intimately included [56, 57]. Regrettably, to our knowledge, it does not appear that there are any further distinct efforts within the profession to enhance the understanding and integration of BPS principles in clinical care. Upon reviewing available online chiropractic colleges’ curricula and course descriptions, there are few indications that BPS related coursework is being presented. If there are any current professional continuing education programs on the topic of the BPS model of care within the chiropractic profession, they have failed to garner widespread attention. Thus, we believe that increased platforms for instruction should be developed and promoted, but also implemented in the delivery of education in chiropractic curricula as well as professional continuing education efforts.

Research into the implementation of the BPS model of care that is specific to chiropractic is scarce at this time. A search of published conference proceedings of the American Chiropractic Colleges Research Agenda Conference (ACCRAC) from the years 2010–2016 produced a total of nine abstract titles associated with a form of psychosocial components of patient care [58–64]. These abstracts included three case reports [58, 62], one pilot study on relaxation therapy [64], two studies investigating fear-avoidance behavior [62, 63], one study regarding education on yellow flags [61], and two studies exploring psychological and psychosocial factors in spine and musculoskeletal disorders [59].

A separate PubMed search using the keywords “chiropractic” and “psychosocial” produced a total of 60 articles. Another PubMed search using the keywords “chiropractic” and “psychological” produced a total of 124 articles. For comparison, a PubMed search using the terms “physical therapy” and “psychosocial” produced a total of 4845 articles, and a PubMed search using the terms “physical therapy” and “psychological” produced a total of 15,402 articles. If the chiropractic profession hopes to be a leader in portal of entry spine related care, it must increase its research focus in this field of study in order to properly identify best practices and the most cost-effective strategies.

As health care transitions toward inter-professional team-based care and emphasizes the BPS model in the management of chronic spine related pain, it is reasonable to expect chiropractors will develop working inter-professional relationships with other team members to increase efforts to promote a unified positive psychosocial influenced method of care. CCE has identified inter-professional education as a required meta-competency for chiropractic programs [54] and some inter-professional efforts to include mental/behavioral health exposure have been made [65, 66]. However, widespread inter-professional educational efforts specific to mental/behavioral health are not known to the authors. Behavioral health providers, such as psychologists and social workers, are further being introduced into team-based settings and the potential synergistic care of behavioral health and chiropractic can potentially be impactful. It is appropriate to expect that initial inter-professional exposure should begin in chiropractic colleges in order to cultivate chiropractic expertise in navigating psychologically informed team based intervention efforts. The need and benefits of health professions education efforts to include inter-professional collaboration has been reported [67, 68] and this avenue would allow for greater opportunities in positive inter-professional training.

Further development of the BPS model within chiropractic education and practice could include:

1. More emphasis on this model within chiropractic education and development of courses or lectures vertically integrated into existing course content.
2. Research on whether BPS principles can be integrated into routine care and doctor-patient encounters such as the report of findings in a successful manner.
3. Research investigating outcomes associated with integrated multi-modal care centered on the BPS model that includes chiropractic intervention.
4. Clinical integration into field-based care through post-graduate education suggesting this model.
5. Continuing education courses that teach chiropractors the basic principles of CBT, MI, and ACT.

## Conclusion

The chiropractic profession has a history rooted in positive BPS model assessment and intervention strategies. However, there remains a component of chiropractic culture that may unintentionally be giving messages that cultivate negative psychosocial behavior in patients. The profession may benefit from increased awareness of routine clinical communication practices, and learn ways of modifying them to be more aligned with BPS principles. It is important that as the landscape of health care shifts toward a BPS paradigm with patient care teams, chiropractic embraces this model and further refines its approach to produce optimal patient outcomes and further establish itself as experts in spine and pain care.

## Abbreviations

ACCRAC: Association of Chiropractic Colleges Research Agenda Conference
ACT: Acceptance and commitment therapy
BPS: Biopsychosocial
CBT: Cognitive behavioral therapy
CCE: Council on chiropractic education
MI: Motivational interviewing
